# ADP-Ribosylation Factor 6 Pathway Acts as a Key Executor of Mesenchymal Tumor Plasticity

**DOI:** 10.3390/ijms241914934

**Published:** 2023-10-05

**Authors:** Ari Hashimoto, Shigeru Hashimoto

**Affiliations:** 1 Department of Molecular Biology, Graduate School of Medicine, Hokkaido University, Sapporo 060-8638, Japan; 2 Division of Molecular Psychoimmunology, Institute for Genetic Medicine, Hokkaido University, Sapporo 060-0815, Japan

**Keywords:** ARF6, AMAP1, PDAC, immune evasion, *KRAS*, *TP53*, PD-1, PD-L1, angiogenesis

## Abstract

Despite the “big data” on cancer from recent breakthroughs in high-throughput technology and the development of new therapeutic modalities, it remains unclear as to how intra-tumor heterogeneity and phenotypic plasticity created by various somatic abnormalities and epigenetic and metabolic adaptations orchestrate therapy resistance, immune evasiveness, and metastatic ability. Tumors are formed by various cells, including immune cells, cancer-associated fibroblasts, and endothelial cells, and their tumor microenvironment (TME) plays a crucial role in malignant tumor progression and responses to therapy. ADP-ribosylation factor 6 (ARF6) and AMAP1 are often overexpressed in cancers, which statistically correlates with poor outcomes. The ARF6-AMAP1 pathway promotes the intracellular dynamics and cell-surface expression of various proteins. This pathway is also a major target for *KRAS*/*TP53* mutations to cooperatively promote malignancy in pancreatic ductal adenocarcinoma (PDAC), and is closely associated with immune evasion. Additionally, this pathway is important in angiogenesis, acidosis, and fibrosis associated with tumor malignancy in the TME, and its inhibition in PDAC cells results in therapeutic synergy with an anti-PD-1 antibody in vivo. Thus, the ARF6-based pathway affects the TME and the intrinsic function of tumors, leading to malignancy. Here, we discuss the potential mechanisms of this ARF6-based pathway in tumorigenesis, and novel therapeutic strategies.

## 1. Introduction

Considering the characteristics of tumor progression, crucial factors that determine tumor fate are mutations, which are an inevitable and permanent consequence of life and a selection of clones adapted to the environment. In normal tissues, excluding germ cells, genetic mutations are known to accumulate at a rate of about 15 to 50 per cell per year [1]. Thus, the continuous accumulation of mutations inevitably leads to diversification at the level of single cells in tumor tissues, both in the tumor cell population and in the normal cell population. On the other hand, cell selection during tumor progression is inferred to be a result of the expansion of clones that are selectively favored by intrinsic genetic traits derived from genomics and epigenomics, and extrinsic factors due to the microenvironment to which the cells are exposed. Thus, the genomes of aging normal tissues and cancers are enriched with environmentally adapted gene variants [2]. To date, more than 500 cancer driver genes have been identified in various cancer types [3], which may induce different cancer features and characteristics that are favorable for cancer growth and survival, which involve not only endogenous mechanisms but also interactions with the tumor microenvironment (TME) [4].

Tumor malignancy progresses not only through intrinsic genetic traits, leading to cancer cells acquiring a mesenchymal transcriptional program, abnormal invasive and metastatic activity, and drug resistance, but also by the creation of an immunosuppressive TME. In addition, alterations in the TME, such as an abnormal vasculature, infiltration of various immune cells, hypoxic conditions, and modified composition of the extracellular matrix (ECM), are known to facilitate the selection of several malignant subpopulations within a single tumor mass [5,6]. These various cells within the TME dynamically and mutually transmit information to the tumor cells, and this interactive communication is considered to be essential for promoting tumor malignancy [7]. Various cytokines, chemokines, and growth factors contribute to intercellular communication within the TME. Moreover, ECM remodeling, i.e., changes in ECM architecture and stiffness due to the mobilization of cancer-associated stromal cells, helps reprogram the cancer cell phenotype to promote invasion and metastasis [8]. Such communication is mediated by cell surface receptors that relay stimuli from the microenvironment to intracellular signaling pathways, thus promoting cancer progression [9].

The small GTPases of the RAS superfamily are one of the largest groups of proteins that transmit extracellular signals via transmembrane receptors into the cytoplasm, and act in a variety of biological responses, such as intracellular trafficking, gene expression, cytoskeleton reorganization, and cell survival [10,11]. These small GTPases cycle between an inactive GDP-bound and active GTP-bound state, acting as molecular switches to regulate intracellular signaling networks. In cancer cells, small GTPases are often dysregulated and have been implicated in cancer development, promotion, and progression [12,13]. ADP-ribosylation factor (ARF) is a small GTPase belonging to the Ras superfamily and is evolutionarily the most ancient GTPase; ARF is conserved throughout eukaryotes, including early divergent species, such as *Giardia lamblia*, in which neither Ras family members nor heterotrimeric G proteins are found [14,15]. *Giardia lamblia* is an intestinal parasitic anaerobic eukaryote with a nucleus and a well-developed cytoskeleton, but without typical mitochondria, peroxisomes, and oxidative phosphorylation components, and is presumed to be an amitochondrial-type eukaryote that developed before the existence of mitochondria [16]. This implies that eukaryotic cellular features, such as nuclei and flagella, predate mitochondrial endosymbiosis, suggesting that ARF family molecules have been deeply involved in maintaining cell homeostasis under anaerobic conditions during eukaryotic evolution. In mammals, the ARF family consists of six isoforms, and is classified into three classes based on sequence homology as follows: class I (ARF1–3), class II (ARF4–5), and class III (ARF6) [17]. ARFs of class I and II mainly control vesicular transport between the endoplasmic reticulum (ER) and Golgi [18]. ARF6, the single class III member, is the most divergent from other ARF proteins, primarily localizes to the plasma membrane and intracellular endosomal compartments, and functions in intracellular events related to membrane dynamics, including endocytosis, recycling back to the plasma membrane, and exocytosis, as well as in the organization of the cytoskeleton [19].

In this review, we provide an overview of the recent insights into the intrinsic and extrinsic roles of ARF6 and its associated signaling machinery in tumor progression. We further summarize the novel molecular mechanisms that we have identified to date involving the activation of ARF6-based signaling pathways, tumor development, and malignant transformation, via *KRAS* and *p53* driver mutations. Finally, we address the potential therapeutic strategies against malignant tumors that target this signaling pathway to promote ARF6 activity.

## 2. Functional Roles of the ARF6-Based Pathway in Cancer Malignancy Caused by the Acquisition of Mesenchymal Properties

Small GTPases are a superfamily of enzymes that function as molecular switches and regulate many cellular processes, including vesicle trafficking [10,11,20]. Dysfunction or dysregulation of some small GTPases, such as members of the RAS and ARF subfamilies, has been implicated in cancer development and progression.

**Figure 1:**
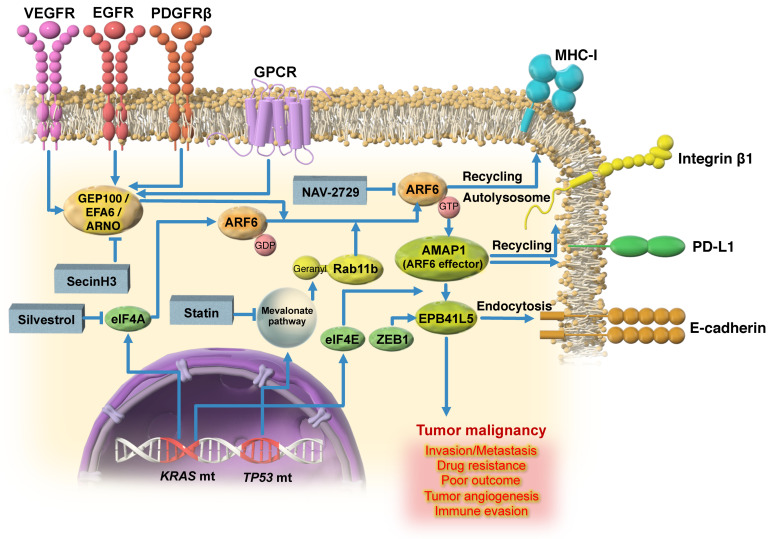
Model of ARF6-mediated tumor malignancy. *KRAS* and *TP53* mutations promote PDAC malignancy via ARF6 and its effector AMAP1 pathway. *KRAS* mutation (mt) promotes the translation of *ARF6* and *AMAP1* mRNA mainly by upregulating eIF4A and eIF4E activity. *TP53* mutation promotes the activation of ARF6 by RTKs via enhancing PDGFR and MVP expression. MVP activity is critical for the geranylgeranylation of RAB11b, which transports ARF6 to the plasma membrane for its activation by RTKs. EPB41L5 is induced during EMT and is associated with the acquisition of mesenchymal properties. The ARF6-AMAP1 pathway promotes the intracellular dynamics of β1 integrin and E-cadherin, and drives tumor cell motility and EMT processes. The ARF6-AMAP1 pathway also enhances PD-L1 dynamics and is closely linked to PDAC immune evasion. In addition, the robust expression and activation of the ARF6-AMAP1 pathway are closely associated with the occurrence of malignancy in tumor cells, including drug resistance, which further leads to poor disease outcomes. Abbreviations: VEGFR, vascular endothelial growth factor receptor, EGFR, epidermal growth factor receptor, PDGFRβ, platelet-derived growth factor receptor β, GPCR, G protein-coupled receptor.

ARF6 is widely expressed in different tissues and organs and regulates protein and lipid trafficking to control cellular function during development and in disease. During tumor invasion and metastasis, the ARF6-specific guanine nucleotide exchanging factor (GEF), GEP100 (BRAG2), is recruited to ligand-activated receptor tyrosine kinases (RTKs), such as epidermal growth factor receptor (EGFR) [21], hepatocyte growth factor receptor (also known as c-MET) [22], platelet-derived growth factor receptor β (PDGFRβ) [23], and vascular endothelial cell growth factor receptor 2 (VEGFR2) [24], and activates ARF6 by mediating the change from ARF6-GDP to ARF6-GTP. Another GEF, EFA6, which binds to GTP-bound Ga12 released from the lysophosphatidic acid (LPA)-mediated G-protein-coupled receptor, also activates ARF6 in clear cell renal cell carcinomas (ccRCCs) [25] (Figure 1). Interestingly, GEP100 activates ARF6 by binding directly to ligand-activated RTKs, and this binding is mediated by the ligand-stimulated interaction between the pleckstrin homology domain of GEP100 and the tyrosine phosphorylation sites of the intracellular domain of RTKs [21]. Indeed, the silencing of *ARF6* or *GEP100* inhibits EGF-induced matrigel invasion by highly invasive human breast cancer cell lines, and further prevents ARF6 activation in the absence of GEP100, indicating that GEP100 contributes to the invasive properties of tumor cells through the regulation of ARF6 activity. Moreover, the simultaneous ectopic expression of GEP100 and ARF6 in epithelial-like breast cancer cells resulted in the EGF-dependent conversion from noninvasive to invasive breast cancer cells. This suggests that the EGFR-GEP100-ARF6 pathway might be significant in the process of acquiring a malignant phenotype in breast cancer cells.

ARF6 activated by GEP100 then recruits its effector AMAP1, which binds to GTP-bound ARF6 in the presence of Mg^2+^ via the ARFGAP domain of AMAP1, an ARFGAP protein [26,27]. AMAP1 colocalizes with ARF6 at invadopodia, and its knockdown efficiently inhibits invadopodia formation and invasive activities in highly invasive human breast cancer cell lines [28]. AMAP1 has several protein interaction motifs, such as the Src homology 3 (SH3) domain and proline-rich domain. The ARF6-AMAP1 pathway is associated with tumor cell motility by promoting actin remodeling and integrin recycling through the binding of AMAP1 to different proteins, such as cortactin and PRKD2 [28,29]. AMAP1 also binds to EPB41L5 [30], a mesenchymal-specific protein induced during the epithelial–mesenchymal transition (EMT), promoting E-cadherin internalization and focal adhesion dynamics to enhance cell motility. The *EPB41L5* gene promoter contains putative binding sites for several EMT transcription factors, including ZEB1, and chromatin immunoprecipitation assays using anti-ZEB1 antibodies demonstrated that ZEB1 binds to the *EPB41L5* gene promoter. In addition, in the Cancer Genome Atlas RNASeq dataset of human primary breast cancers (*n* = 970), high levels of *ZEB1* mRNA statistically correlated with high levels of *EPB41L5* mRNA [31]. These results demonstrate that ZEB1 is essential for induction of the *EPB41L5* gene, and the ARF6-based pathway is linked to mesenchymal properties.

Mutations in *TP53* not only lead to the loss of wild-type p53 functions, but also exert oncogenic gain-of-function activities in cancer cells by conferring new abilities on the mutant protein. These p53 mutants are frequently seen in human tumors and are associated with the promotion of cancer progression [32]. In breast cancer, mutant p53 has been shown to enhance tumorigenesis via the metabolic mevalonate pathway (MVP), which is associated with protein prenylation, involving attachment of either a farnesyl or a geranylgeranyl isoprenoid to a protein [33].

Most small GTPases require lipid modifications, such as geranylgeranylation, farnesylation, myristoylation, and palmitoylation, for their function. Geranylgeranylation and farnesylation are classified as MVP-mediated prenylations. A strategy to inhibit the MVP as a cancer treatment was proposed by Brown and Goldstein [34]. Statins are inhibitors of hydroxymethylglutaryl-CoA reductase, which is the rate-limiting enzyme of MVP, and were originally developed to lower cholesterol levels in patients with hypercholesterolemia. Importantly, upregulation of the MVP by *TP53* mutations promotes the activation of ARF6 [22]. The underlying mechanism involves the trafficking of ARF6 to the plasma membrane via lipid modification of Rab11b by the MVP enzyme geranylgeranyl transferase II (GGT-II), where it is activated by receptor tyrosine kinases via an external ligand (Figure 1). The inhibition of GGT-II not only blocked tumor invasion and metastasis, but also reduced tumor cell resistance to chemotherapeutic agents in cells overexpressing components of the ARF6-based pathway. The overexpression of components of the ARF6-based mesenchymal pathway and enhanced MVP activity also correlated with poor prognosis in breast cancer patients [22]. Furthermore, the MVP inhibitor statins were found to inhibit TGFβ1-induced ARF6 activation and invasion in breast cancer cells, and also reduced tumor cell resistance to chemotherapeutic agents. Thus, the ARF6-based mesenchymal pathway appears to promote tumor malignancy of breast cancer in cooperation with MVP activity.

Activating mutations in *KRAS*, as well as *TP53* mutations, are the most frequently occurring mutations in human cancers, and are particularly prominent in human pancreatic ductal adenocarcinoma (PDAC). These *KRAS* and *TP53* mutations co-occur in approximately 70% of patients with PDAC, and act as major drivers affecting tumor progression and prognosis. Importantly, mutant *KRAS* acts in a eukaryotic translation initiation factor (eIF) 4A-dependent manner to enhance the translation of ARF6 mRNA, which has a quadruplex structure in the 5′-untranslated region [35] and upregulates the expression of the ARF6 protein. It has also been demonstrated that ARF6 is involved in the regulation of aerobic glycolysis via c-Myc regulation in PDAC with *KRAS* mutations [36]. This suggests that the *KRAS*-mutation-mediated upregulation of ARF6 may contribute to the progression of malignancy by maintaining high metabolic and proliferation rates in PDAC. Moreover, *KRAS* mutations promote the eIF4E-dependent translation of AMAP1 mRNA containing 5′-terminal oligopyrimidine-like sequences by upregulating mTORC1 [37,38]. Thus, the high protein expressions of ARF6 and its downstream effector, AMAP1, are the major targets of the *KRAS*/*TP53* mutations, and may be a crucial pathway that drives PDAC invasion and metastasis, as well as PD-L1 recycling and immune evasion, as described later, to enhance the progression of malignant mesenchymal tumors (Figure 1).

## 3. ARF6 Pathway Conferring Mesenchymal Properties Causes Drug Resistance and Modulation of the TME

EMT is a cellular program that often occurs in both normal and pathological processes. It is essential for embryogenesis and wound healing, and plays important roles in the malignant progression of many types of carcinomas [39]. In the case of neoplasia, EMT is caused by a variety of factors and is associated with increased tumor-initiating and metastatic potential and a higher resistance to elimination by some therapeutic regimens [40,41,42,43,44]. It has also been shown that the acquisition of mesenchymal properties by cancer cells increases their resistance to elimination by cells of the adaptive immune system that are present in the tumor-associated stroma, suggesting that EMT is also involved in the regulation of anti-tumor immunity [45,46].

From recent studies, EMT in cancer is thought to be a plastic process in which cells convert between epithelial/mesenchymal (E/M) hybrid states with both epithelial and mesenchymal characteristics, also called epithelial–mesenchymal plasticity (EMP; ref. [47]). The role of the E/M hybrid state has attracted attention because it has been shown not only to confer drug resistance, but also to modify the immune system to support cancer invasion and metastasis [39,48]. Recent cancer studies using single-cell RNA sequencing have shown that the EMP gene program appears to be conserved across cancer types, but the constituent genes are variable and correlate with the tissue of origin [49,50,51]. Importantly, these E/M hybrid states result in immune evasion of the tumor, with decreased infiltration of cytotoxic T-lymphocytes (CTLs) and increased infiltration of immunosuppressor cells [46,52,53,54]. As mesenchymal carcinoma cells acquire increased resistance to both chemotherapy and immunotherapy, and even a small fraction (10%) of hybrid E/M cells cause immune evasion in tumors [46], it appears to be important to understand how to enhance plasticity within the tumor through EMT programs.

As mentioned above, our previous studies have shown that breast cancer cells with mesenchymal properties utilize the ARF6 pathway for invasion and metastasis [21,22,26,28,29]. Moreover, the ARF6 pathway appears to be a mesenchymal-specific pathway that includes the mesenchymal-specific protein EPB41L5 as its integral component [31], and that EPB41L5 is mainly induced by ZEB1, an EMT-associated transcription factor [55,56,57,58]. ZEB1 has also been shown to be involved in conferring drug resistance in pancreatic cancers [59,60,61]. Interestingly, AMAP1 and EPB41L5, which are components of the ARF6-based mesenchymal pathway, also play an important role in promoting drug resistance [31]. Generally, the molecular mechanisms proposed to underly drug resistance include the regulation of stem cell properties, altered expression of anti-apoptotic proteins, decreased drug uptake caused by altered influx transporters, and chromatin remodeling driven by EMT-transcription factors (TFs) [62,63,64]. The TME, which is a site of dynamic and constant interaction among tumor cells, is also known to affect therapeutic resistance [65]. In addition, tumor-derived exosomes have also been shown to promote EMT induction, as well as therapeutic resistance [66]. Recently, mathematical methods have also been used to study EMT and its potential involvement in drug resistance. For instance, a computational approach named Markov-affinity-based graph imputation of cells, developed to recover missing gene expressions in single cell data, suggested that drug resistance in cancer cells is maximized at intermediate levels of activation of the EMT program [67]. Thus, although intriguing findings on the correlation between EMT and drug resistance have been obtained, the specific mechanisms involved are still unclear and may differ among different cancer types. Analysis of the precise molecular mechanisms of how the ARF6 pathway, which confers mesenchymal properties, contributes to drug resistance may be important in the development of cancer therapeutic strategies.

Through many studies on cancer development, it has become evident that tumor cells in early-stage carcinomas are in an epithelial-like state, and gradually acquire mesenchymal properties as the tumor progresses. Carcinoma cells acquiring mesenchymal properties have been shown to be more resistant to elimination by cells of the adaptive immune system in the tumor-associated stroma [45,46], highlighting the regulatory mechanism of EMT-triggering anti-tumor immunity. When EMT is induced in carcinoma cells, mesenchymal neoplastic cells are known to respond by modifying the properties and activity of various types of cells attracted to the stroma, particularly innate and adaptive immune cells that affect tumor progression [68,69,70]. Several reports have suggested that cancer cells expressing EMT-associated markers affect immune cells with anti-tumor functions, such as cytotoxic CD8^+^ T-cells, and immunosuppressive regulatory cells, such as Treg cells, and thus contribute to the generation of an immunosuppressive TME [71,72]. The human breast cancer cell line MCF-7 causes T-cell reactivity when cocultured with antigen-specific T-cell clones. However, MCF-7 cells expressing SNAIL, an EMT-TF, has been shown to substantially impair T-cell function when cocultured with these T-cell clones [73]. It has also been reported that SNAIL-expressing melanoma cells induce immunosuppressor Treg cells and are resistant to immunotherapy [71]. Although these studies clearly demonstrated a link between the EMT program and impaired immune surveillance, the detailed mechanisms that confer resistance to immune attack on mesenchymal carcinoma cells remain to be investigated. One study has shown that the induction of EMT attenuates the cytolytic activity of CD8^+^ T-cells by inducing autophagy in target cells, in addition to inducing the release of immunomodulatory cytokines and chemokines [72]. In addition, activation of the EMT program weakens T-cell priming by altering the formation of immunological synapses between cancer cells and T-cells. The altered cell surface expression of immunomodulatory markers causing them to escape from immune surveillance is another mechanism of immune evasion. For example, in breast cancer, the induction of EMT is known to attenuate the cell surface display of major histocompatibility complex (MHC) class I molecules, which are required for antigen presentation to T-cells [74]. Thus, by downregulating the expression of MHC class I molecules, tumor cells can circumvent the cytolytic function of T-cells [75,76]. In addition, EMT has been shown to induce the expression of PD-L1, which triggers T-cell exhaustion, by cancer cells [46,77,78]. Furthermore, EMT signatures in non-small cell lung cancer (NSCLC) patients have been shown to correlate with the elevated levels of multiple immune checkpoint proteins and the presence of Treg cells within the primary tumor [79]. Thus, it is noteworthy that carcinoma cells that have acquired mesenchymal properties are associated with the creation of an immunosuppressive TME, by regulating the activity of stromal constituents, particularly immune cells, through various processes.

Notably, it has been indicated that the ARF6 pathway is linked to the promotion of PD-L1 dynamics [23] and MHC-I recycling [80] via its activation by external ligands, resulting in reprogramming to the immunosuppressive TME. In human and mouse PDAC, MHC-I is degraded through an autophagy-dependent mechanism to cause immune evasion [81]. ARF6 plays an important role in the regulation of autophagy, particularly autophagosome formation [82,83,84]. In PDACs with a high expression of ARF6, ARF6-mediated autophagy activity is high, which may result in the suppression of cell surface MHC-I molecules. Therefore, it will be very interesting to study how this linkage regulates immunomodulatory markers and induces immune evasion. A detailed investigation will be required on how activation of the ARF6-based pathway affects the number and function of immune cells in the TME and their response to various immunotherapies, and consequently how the ARF6-based pathway modulates anti-tumor immunity. Elucidating the molecular mechanisms underlying immunosuppression via EMT-mediated mesenchymal properties may lead to the identification of novel immunomodulatory markers that can predict tumor progression and responses to immunotherapy, and to advances in the development of new therapeutic strategies.

## 4. ARF6 Pathway Controls Immunomodulatory Molecules

### 4.1. ARF6 Functions as a Regulator of PD-L1

**Figure 2:**
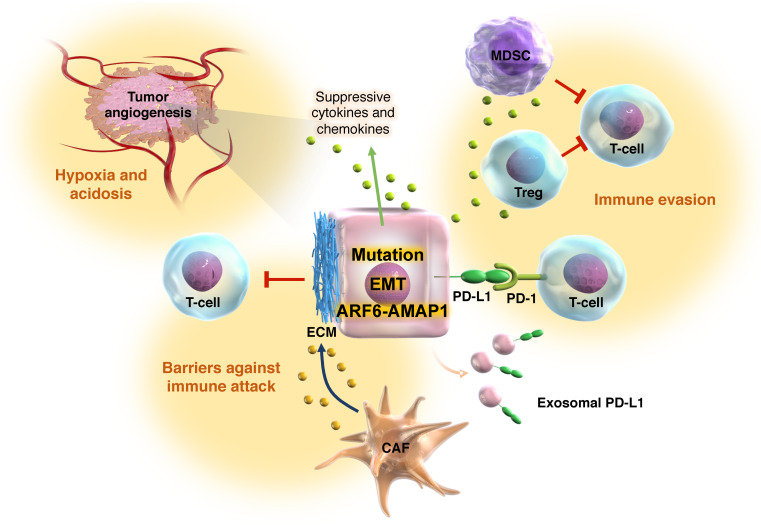
Effects of the ARF6-AMAP1 pathway on the TME. In PDAC, the ARF6-AMAP1 pathway is upregulated by driver mutations and drives the cancer mesenchymal program. Tumors with mesenchymal properties have an immunosuppressive tumor environment. Indeed, the ARF6-AMAP1 pathway is closely linked to immune evasion and fibrosis, which are well-known barriers to immune attack against cancer cells. Moreover, the ARF6-AMAP1 pathway also contributes to tumor angiogenesis, leading to cancer malignancy. Abbreviations: MDSC, myeloid derived suppressor cells; Treg, regulatory T cell; CAF, cancer-associated fibroblast.

The ARF6 pathway is associated with the recycling of the immune checkpoint molecule PD-L1, as well as cell surface components, such as β1-integrin and E-cadherin [23,29,84]. PD-L1 is overexpressed in tumor cells as an immunosuppressive factor, and inhibits the function of CTLs, contributing to the immune evasion of cancer [85]. Interestingly, PDGF-stimulated activation of the ARF6-AMAP1 pathway regulates the intracellular dynamics of PD-L1 in PDAC, suggesting that the cooperation of *KRAS* and *TP53* mutations, which enhance ARF6 and AMAP1 expression, may be responsible for promoting PD-L1 dynamics. Silvestrol (an eIF4A inhibitor) and simvastatin (an inhibitor of the MVA metabolic pathway), which inhibit ARF6 expression and activity, have been shown to cause the diffuse distribution of PD-L1 throughout the cell surface [23]. In 2017, the ckLF-like MARVEL transmembrane domain contains 6 (CMTM6) were identified as key proteins that regulate PD-L1 stability. Two groups showed that CMTM6 colocalizes with PD-L1 at the plasma membrane and recycling endosomes, preventing PD-L1 from degradation by lysosomes [86,87]. CMTM6 has been shown to have oncogenic properties and is highly expressed in a variety of tumors, including colorectal cancer [88], hepatocellular carcinoma [89,90], and kidney cancer [91], and is closely associated with the poor prognoses of patients [92]. Therefore, CMTM6 is a promising biomarker for predicting the efficacy of immunotherapy [93,94]. Extracellular vesicles containing exosomes secreted by tumor cells are also known to carry immunosuppressive molecules, such as PD-L1, to the surrounding immune cells and to contribute to tumor progression [95,96,97,98] (Figure 2). CMTM6 has been shown to contribute to the immunosuppressive state of the TME through exosome-mediated shuttling [99].

Interestingly, ARF6 has also been shown to regulate the production of exosomes. Indeed, it has been reported that the inhibition of ARF6 in MCF-7 cells leads to reduced exosome release, and that ARF6 activity is required for exosome production [100]. Therefore, it would be very meaningful to investigate how the ARF6 pathway, which plays crucial roles in the regulatory function of membrane traffic and exosome secretion, is associated with CMTM6, which is involved in the regulation of PD-L1 recycling back to the cell surface. If the ARF6 pathway and CMTM6 function cooperatively to induce an immunosuppressive microenvironment, their cooperation could be a potential new target that will benefit immunotherapy.

### 4.2. ARF6 Functions as a Regulator of MHC-I

Antigen presentation by MHC proteins plays an important role in adaptive immunity. MHC class I molecules bind peptide fragments of intracellular proteins and present them to the cell surface, and their expression on the cell surface is important for immune recognition and defense against infections and cancer. One of the functions of ARF6 is to regulate the recycling of MHC class I molecules on the cell surface [101]. MHC class I molecules are synthesized in the ER and then transported to the cell surface through the secretory pathway. After antigen presentation, MHC class I molecules have been shown to be internalized by the clathrin-independent endocytosis pathway (CIE) and dynamin [101,102], and some of the MHC class I molecules are then recycled back to the cell surface. After endocytosis of MHC class I molecules, vesicles fuse with early endosomes, and some MHC class I molecules proceed to late endosomes and lysosomes, where they are degraded. Analysis using HeLa cells has demonstrated that when MHC class I molecules are endocytosed, approximately half of them are degraded in late endosomes and half of them are recycled [101].

ARF6 has been shown to regulate the recycling of MHC class I molecules by promoting their retrieval from endosomes and their transport to the cell surface. Indeed, expression of the inactive mutant Arf6T27N in HeLa cells has been shown to inhibit the recycling of MHC class I molecules [101,102,103]. In contrast, expression of the constitutively active mutant Arf6Q67L results in a halt in the endocytosis of MHC class I molecules, because over time, an enlarged vacuolar structure containing CIE cargo proteins, including MHC I molecules, accumulate and trap membranes [104]. Therefore, ARF6 might play an important role in antigen presentation by controlling the recycling of MHC class I molecules. Overall, the mechanism by which ARF6 regulates MHC class I expression is complex, and likely involves multiple pathways involving Rab and other molecules. As mentioned above, in PDAC, MHC-I molecules are degraded via an autophagy-dependent mechanism, inducing immune evasion [81]. Therefore, in some carcinomas, the ARF6-mediated promotion of autophagy activity may result in the loss of cell surface MHC-I molecules. Understanding these mechanisms may provide new targets for developing therapies to enhance immune recognition and to improve the prognosis of cancer patients, as well as those with infectious diseases.

### 4.3. Functional Roles of the ARF6-AMAP1 Pathway in Immune Evasion

Notably, we previously demonstrated that the high expression and activation of the ARF6-AMAP1 pathway promote immune evasion as well as invasion and metastasis in PDACs with *KRAS*/*TP53* mutations as driver genes [23]. In this analysis, using KPC cells isolated from KPC (LSL-Kras[G12D/+]; LSL-Trp53[R172H/]; Pdx-1-Cre) mice, an established model of human PDAC, we found that silencing the expression of ARF6-AMAP1 pathway components does not affect KPC cell growth in immunodeficient mice, but robustly inhibits growth in syngeneic immunocompetent mice [23]. This study provides new insights into how the ARF6-AMAP1 pathway is closely associated with the immune evasion of PDAC cells, although the precise mechanism remains to be elucidated.

Tumor immune evasion involves a variety of factors, involving metabolic intercommunication or competition between tumor cells and the stroma [105,106], acidosis of the TME [107], and even tumor fibrosis [108] (Figure 2). Dense fibrosis is a hallmark of PDAC and often represents a dual aspect of cancer biology and therapeutics [109]. Reducing fibrosis by depleting cancer-associated fibroblasts in the PDAC mouse model leads to immunosuppression in the TME and reduced mouse survival [110]. On the other hand, the reduction in fibrosis may be a prerequisite for improved checkpoint immunotherapy, by enabling immune cells to access tumor cells more efficiently [111]. In fact, the analysis of human lung cancer tissue has confirmed that fibroblasts and collagen accumulate in the tumor matrix, inhibiting the interaction between T-cells and tumor cells [112,113].

In Japanese clinical specimens of PDAC, we found that high expression of the AMAP1 protein statistically correlated not only with fibrosis progression but also with the high expression of PD-L1 [114]. Based on our analysis, whereas the upregulation of PD-L1 expression and dynamics, as well as the promotion of fibrosis, may contribute to the mechanism by which the ARF6-AMAP1 pathway induces tumor immune evasion, there may be some other unidentified mechanisms by which this pathway is driven.

The effects of EMT-mediated immune evasion have also been observed in various cancers. For example, breast cancers in the epithelial state are heavily infiltrated with CD8^+^ T-cells, whereas breast cancers in the mesenchymal state contain a large number of Treg cells and tumor-associated macrophages (TAMs) [71]. It has also been reported that only tumors in the epithelial state are suppressed by anti-CTLA4 immune checkpoint inhibitor (ICI) therapy, whereas tumors in the mesenchymal state are resistant to this therapy [46]. As described above, the resistance of cancer cells in the mesenchymal state to attack from CTLs was also demonstrated in human breast cancer cells [73] and melanoma cells expressing SNAIL [71]. These studies clearly indicate the association between EMT and immune surveillance, and hence the mechanism of immune evasion by the ARF6-AMAP1 pathway, which is driven by mesenchymal properties, is a challenge to be solved. Our study highlights targeting of the ARF6-AMAP1 pathway as a potential strategy to enhance immune recognition and response to pancreatic cancer, and to overcome the immune evasion mechanisms used by tumor cells.

## 5. ARF6 Pathway Is Associated with Pathological Angiogenesis

Angiogenesis is the process by which new blood vessels are formed, primarily by sprouting from existing vessels [115]. Endothelial cells (ECs) lining the luminal surface of blood vessels are typically quiescent, but can be activated during development, wound healing, and disease to be involved in the process of angiogenesis [116]. When a tumor is less than 1 mm in diameter, there is little formation of blood vessels, and the cells rely primarily on permeation to acquire nutrients [117,118]. As growing tumors require nutrients to meet their demands, cancer cells favorably promote angiogenesis by releasing proangiogenic factors, which in turn stimulate the growth of new blood vessels. Tumor vasculature is marked by an abnormal vessel structure with perfusion deficits, resulting from an imbalance between pro- and anti-angiogenic signaling within tumors [119]. These abnormalities in vascular structure result in an inefficient supply of oxygen and nutrients to tumor cells and obstruction in the removal of metabolic waste, which in turn lead to hypoxia and acidosis in the TME. Tumor-hypoxia-induced responses have been reported to alter gene expression, inhibit apoptosis, and promote autophagy [120,121], EMT, malignant progression, distant tumor metastasis [122,123], and angiogenesis [124,125,126]. Hypoxia is also known to affect the immune system through a variety of pathways, contributing to reduced anti-tumor responses [127,128]. Thus, hypoxia in tumors caused by the abnormal formation of blood vessels may make the TME favorable for tumor expansion and malignant progression, leading to immune escape, the ineffectiveness of anti-tumor therapies, and eventually tumor recurrence. Recent single-cell analyses have shown that ECs throughout tissues demonstrate unique molecular signatures, and that the interplay between tumor ECs and immune cells induces the tissue-specific immunomodulatory function of ECs [129]. This presents a molecular rationale for combining tumor EC targeting with the immune checkpoint blockade (ICB) for cancer therapy.

**Figure 3:**
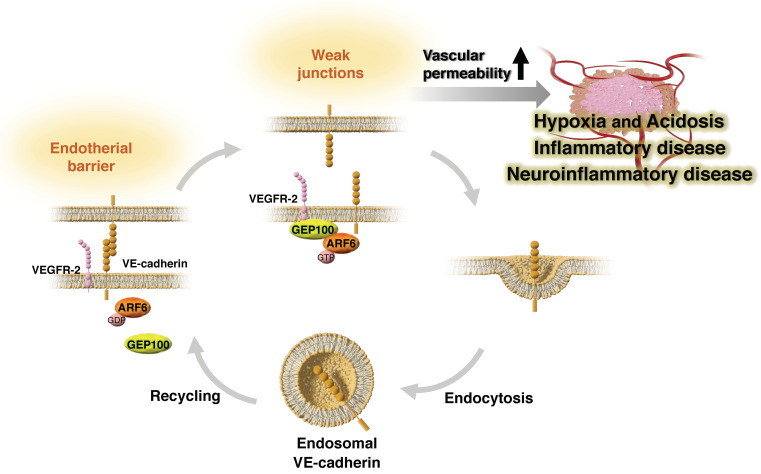
Schematic model of ARF6 pathway-mediated vascular plasticity. The vascular endothelial barrier is controlled by VE-cadherin-mediated endothelial cell–cell junctions. VEGF stimulation activates VEGFR2, which in turn activates several signaling factors, including ARF6, which dissociates VE-cadherin, releases endothelial cell–cell junctions, and increases vascular permeability. The ARF6-based pathway is involved in the reorganization of cell–cell junctions based on VE-cadherin as well as endothelial cell migration/tubular network formation, thus contributing to the regulation of vascular permeability. Abbreviations: VEGFR-2, vascular endothelial growth factor receptor-2.

Interestingly, components of the ARF6-based pathway are highly expressed in human umbilical vein ECs (HUVECs), and have been found to be essential for angiogenic activities, such as cell migration and tubular formation, as well as for enhancing cell permeability and VE-cadherin endocytosis in vascular endothelial growth factor (VEGF)-stimulated HUVECs [24]. VEGF, a major factor involved in angiogenesis [130,131,132,133], was originally discovered as a vascular permeability factor [134], and VEGF signaling was found to promote EC proliferation, migration, and permeability, causing vascular leakage [135]. We found that GEP100 physically binds to the VEGF-activated receptor VEGFR2 to activate ARF6, which in turn recruits AMAP1. This GEP100-ARF6-AMAP1 pathway is essential for VEGF-induced EC migration and tubular formation as well as VEGF-induced VE-cadherin endocytosis and enhanced cell permeability (Figure 3). Indeed, a cell-permeable peptide that inhibits the downstream signaling of the ARF6-AMAP1 pathway was effective in suppressing laser-induced choroidal neovascularization [136,137] in mice, a model of choroidal neovascularization, which is a major cause of severe vision loss in patients with age-associated macular degeneration [138], indicating that the ARF6-AMAP1 pathway may also be involved in pathological angiogenesis. This ARF6-based pathway may be associated with at least the sprouting process of angiogenesis, as it is involved in the reorganization of cell–cell junctions based on VE-cadherin as well as cell migration/tubular network formation activities.

ECs are important mediators of the inflammatory response, as they act as a barrier for inflammatory cells to migrate from the blood to the tissues. Cytokines involved in immune defense can also have a destructive effect on endothelial cell–cell interactions and destabilize the endothelial barrier, resulting in vascular permeability [139,140,141]. Myeloid differentiation factor 88 and ARF6 activation via ARF nucleotide binding site opener (ARNO) have been shown to be involved in vascular permeability induced by interleukin (IL)-1β [142]. Furthermore, SecinH3, which inhibits ARFGEFs, such as ARNO, enhances vascular stability and substantially improves outcomes in animal models of inflammatory arthritis and acute inflammation. In addition, in a mouse model of diabetic retinopathy, endothelium-specific ablation of ARF6 protected against vascular leakage by reducing VEGFR2 signaling capacity [143]. Mechanistically, the activation of ARF6 by ARNO has been shown to be involved in VEGF-induced VEGFR2 internalization, and GEP100-dependent activation of ARF6 is required for the maintenance of VEGFR2 protein levels and receptor recycling. Different GEFs acting at two regulatory points during the intracellular trafficking of VEGFR2 are known to be involved in the activation of ARF6, exerting different effects on the signaling pathway through endocytosis and recycling. NAV-2729, a small molecule inhibitor of ARF6, was also shown to abrogate vascular permeability and pathological angiogenesis in a rat model of diabetic retinopathy [143]. These findings suggest that ARF6 inhibition may be an effective therapeutic approach for diabetic retinopathy by inhibiting pathological VEGF signaling.

Vascular leakage is not only associated with inflammatory diseases, such as diabetic retinopathy and arthritis [142,143,144,145,146], but also other neuroinflammatory diseases, including Alzheimer’s disease, Parkinson’s disease, and Huntington’s disease [147,148,149,150,151]. Although it has been noted that the underlying mechanisms of vascular leakage in inflammatory diseases largely involves the disruption of adherens junctions and tight junctions [142,143,144,145,146], recent studies on neuroinflammatory diseases suggest that the endothelial-to-mesenchymal transition (EndoMT) may also be a major factor [152,153]. EndoMT is the process by which ECs undergo a series of molecularly driven events that cause them to take on a mesenchymal cell phenotype [154]. In EndoMT, the expression of mesenchymal markers such as N-cadherin is enhanced, and the expression of EC adhesion molecules, such as VE-cadherin, is suppressed [155,156]. EndoMT is known to be a key player in the inflammatory process [157], resulting in microvascular reorganization through cytoskeletal remodeling and increased endothelial permeability [154]. Recently, it was shown that EndoMT occurs in the central nervous system (CNS) and plays a significant role in the breakdown of blood-CNS barrier (BCNSB) function [153]. EndoMT triggered by IL-1β-stimulated signaling activates ARF6, which in turn induces the activin-receptor-like kinase-SMAD1/5 pathway. In addition, pharmacological treatment with an ARF6 inhibitor (NAV-2729) blocked ARF6 activation in an experimental autoimmune encephalomyelitis mouse model, enhancing BCNSB function, reversing EndoMT, and reducing disease progression. The ARF6-based pathway plays a significant role in vascular ECs, may affect the CNS as well as tumor malignancy, and targeting ARF6 or components of its downstream signaling pathways may have extensive therapeutic implications for many diseases.

## 6. Anti-Cancer Therapeutic Strategy Based on Targeting of the ARF6-AMAP1 Pathway

In recent years, immunotherapy, which treats cancer by regulating the immune system, has been the focus of much research [105,158,159]. In particular, research is being intensively conducted to further extend the survival period of cancer patients by identifying effective combinations of drugs for ICB therapies. However, despite multifaceted research on the treatment of PDAC, there is still no effective ICB-based therapy or biomarker for this cancer type [84].

Our series of studies demonstrated that ARF6 and its downstream effector AMAP1 are often overexpressed in different cancers, including PDAC, and this overexpression is statistically correlated with the poor prognosis of cancer patients [21,28,160,161,162]. The ARF6-AMAP1 pathway controls the intracellular dynamics of β1-integrin, E-cadherin, and PD-L1, and is closely linked to the phenomenon of tumor cell malignancy, such as the promotion of invasion, metastasis, and immune evasion [23,29,31]. Furthermore, we found that activation of the MVP is crucial for the activation of ARF6 by external ligands and statins, which are inhibitors of the MVP, and furthermore, GGT-II inhibitors were found to inhibit the ARF6-based pathway [22]. We also found that oncogenic *KRAS* mutations promote mRNA translation processes dependent on eIF4A and eIF4E, leading to the overexpression of ARF6 and AMAP1 proteins, respectively (Figure 1). eIF4A is an RNA helicase required to unwind the secondary structure of mRNAs containing G-quadruplex (G4) structures to initiate the elongation step of translation [163], and is known to be inhibited by silvestrol [35,164]. ARF6 mRNAs in both humans and mice contain the G4 structure [23], and we previously demonstrated that silvestrol markedly reduces the levels of the ARF6 protein in PDAC cells with *KRAS* mutations [23]. Notably, a recent study using transcriptome-scale ribosome footprinting in PDAC detected eIF4A-dependent mRNAs, such as those of *KRAS* and its signaling molecules, *PI3K*, *RAC2, RALA*, *MYC*, *MET*, and *YAP1* [165].

In the experiments evaluating the anticancer effects of ICI using a tumorigenesis model based on transplantation of KPC cells into syngeneic mice, we have found that the synergistic effect of anti-PD-1 antibody and AMAP1 silencing demonstrates a significant tumor suppression effect. This effect surpasses the tumor inhibition observed with the administration of anti-PD-1 antibody alone [166]. Next, we tested the effect of silvestrol and found that silvestrol alone had no anti-tumor effect on PDAC cells, but silvestrol had a robust synergistic effect with the anti-PD-1 antibody in intact KPC cells, as seen in AMAP1-silenced cells. These results suggest that inhibition of the ARF6-AMAP1 pathway on its own alleviates some of the immune evasiveness of KPC tumor cells, but as we have previously shown [23], inhibition of this pathway together with anti-PD-1 antibody treatment results in therapeutic synergy. Silvestrol is not yet applicable to humans, but with attempts to improve eIF4A inhibitors, combination therapy with ICIs may be the key to effective immunotherapy.

Mutant p53 is known to support tumorigenesis via the MVP, and we found that activation of the MVP by mutant p53 promotes ARF6 activation via GGT-II and Rab11b [22]. We previously demonstrated that simvastatin, as well as silvestrol, and GGT-II silencing inhibit the invasion of breast cancer cells overexpressing the ARF6-based pathway, and also decrease the cell surface expression of PD-L1 in PDAC cells. Interestingly, statins, particularly simvastatin, were shown to function as adjuvants that strongly induce immune responses [167]. Hence, it was suggested that the modulation of post-translational geranylgeranylation is an important mechanism for altering immune functions. Indeed, simvastatin is known to reduce the geranylgeranylation of small GTPases, which stops the maturation of intracellular endosomes and enables many antigens to be presented for a longer time than untreated cells. As a result, strong antigenic stimulation occurs for a longer period of time, indicating an enhanced immune response. Furthermore, this study shows that the injection of cancer cells and simvastatin into mice, followed by treatment with anti-PD-1 antibodies, strongly inhibits cancer growth [167]. In addition, some cancer patients taking statins have reported significantly improved cancer outcomes [168,169]. Recently, inhibition of the MVP with statins in ARID1A (encoding a subunit of the SWI/SNF complex)-inactivated clear cell ovarian carcinoma was shown to induce inflammasome formation and pyroptosis synergistically with anti-PDL-1 to suppress tumor growth [170]. The induction of pyroptosis in tumor cells is known to promote the infiltration of immune cells, such as CD8^+^ T-cells [171], suggesting that the inhibition of MVP by statins may also enhance anti-tumor immunity in the TME. These studies suggest that the combination of statins and ICIs may be effective as a means of enhancing anti-tumor immunity, but the characteristics of the tumors that benefit must be determined.

## 7. Conclusions and Prospects

Since the beginning of the 21st century, as cancer genome research progressed, driver mutations of cancer were discovered one after another, and targeted drugs developed against these mutations were found to be highly effective, creating an uplifting atmosphere that cancer could be conquered through genome research. However, subsequent research has shown that no matter how large the effect, cancer clones eventually emerge that are resistant to the targeted drugs, and the idea that targeted drugs alone can cure the disease has faded. While genetic risk factors including racial and ethnic differences must be considered, the main reason for this lack of therapeutic success is the vast heterogeneity of mutations in the genome of cancer cells, and the acquisition of phenotypic plasticity through extrinsic effects of the TME, which may be the main cause of therapeutic resistance.

On the other hand, the close association between treatment resistance and invasiveness/metastatic potential is evident in the fact that large metastatic or clinical stage IV disease is almost incurable, with 5-year survival rates ranging from 5% to 30%. Treatment applies additional selective pressure on cancer cells that have acquired mesenchymal traits, leading to the generation of tumor subclones with treatment-resistant mutations, and the induction of an inflammatory response that can promote lineage formation. The accumulation of much medical and biological knowledge on the basic mechanisms of therapeutic resistance and the acquisition of invasive and metastatic abilities will provide many opportunities to further improve therapeutic outcomes. However, without a systematic understanding of these mechanisms, the results of outcomes will be the same as with conventional chemotherapy, targeted therapy, immunotherapy, and other therapies. Importantly, increasing our understanding of the overall molecular mechanisms of cancer resistance and the acquisition of invasiveness and metastatic potential will identify the actual key factors and their association with relevant microenvironmental mediators.

The ARF6-AMAP1 pathway may function together with the intrinsic plasticity as well as diversity of the extrinsic TME in cancer, leading to tumor malignancy. Particularly, in PDAC, the expression of ARF6 and AMAP1 is driven by the representative driver mutation of *KRAS*, and ARF6-AMAP1 activity is enhanced through activation of the MVP by mutant *TP53*, contributing not only to invasion and metastasis but also to PD-L1 dynamics and immune evasion (Figure 1). The ARF6-based pathway is also involved in the dynamics of VE-cadherin, contributing to angiogenesis and vascular permeability. Thus, the ARF6-based pathway also extrinsically plays an essential role in vascular endothelial cells and is involved in the pathogenesis of malignant tumor progression.

The ARF6-AMAP1 pathway is a promising druggable target to reduce tumor malignancy. Studies on drugs targeting ARF6 and its upstream or downstream molecules have demonstrated that the eIF4A inhibitor silvestrol, which blocks the translation of *ARF6* mRNA, and the MVP inhibitor statin, which blocks ARF6 activity, inhibit ARF6-based tumor malignancy. The ARFGEF inhibitor SecinH3 and the ARF6 inhibitor NAV-2729 have potential for cancer therapy, as they enhance vascular permeability and have shown efficacy in various animal pathological models. In addition, promising compounds targeting mutant KRAS proteins are being developed and have shown promising results in clinical trials [172].

Therefore, ARF6 and its effectors play pivotal roles in cancer progression and are potential novel biomarkers and drug targets for malignant cancers. In addition, elucidation of the detailed molecular mechanisms of tumor malignancy involving ARF6 signaling may also provide clues to new therapeutic targets and strategies.

There has recently been a rapid advance in single-cell and spatial technologies, as well as computational systems biology, which will enable us to study evolving clonal dynamics and to understand the role of the TME at an unprecedented resolution and scale. A growing number of technologies are being used to analyze epigenetic marks, proteins, and metabolites at the single-cell level and with high spatial resolution [173,174]. With these technological advances, the challenge of the single-cell era of cancer biology is to create robust, reproducible, and transparent ways to analyze, interpret, and share the growing amount of “big data”. Education on algorithms and computational methods are needed, as well as the development of experimental approaches to rigorously test hypotheses generated from tumor profiling and to define molecular mechanisms and link these to novel therapeutics. Thus, it is imperative to strengthen the integration of life sciences, physical sciences, engineering, computational science, and artificial intelligence to create the next generation of cancer therapeutics.

## Data Availability

Not applicable.
